# Supplementary material for: A Likelihood Approach for Real-Time Calibration of Stochastic Compartmental Epidemic Models
Source: PLoS Comput Biol. 2017 Jan 17;13(1):e1005257. doi: 10.1371/journal.pcbi.1005257 (PMC5240920; doi:10.1371/journal.pcbi.1005257)
Supplement: S1 File — (TAR.GZ) [file pcbi.1005257.s014.tar.gz › HSPH_Online-SI-Revision/PF08/n10-mild/n10-mild_PF_table8.pdf]

| | | | | | | | | | | | | | | | | | | | | | | | | | | | |
|---|---|---|---|---|---|---|---|---|---|---|---|---|---|---|---|---|---|---|---|---|---|---|---|---|---|---|---|
| 1 | R0 | {1.25046, 6.80278} | {1.53197, 19.7937} | {{1.49302, 19.4228}, {1.55845, 20.}} | {22.5129, 190.965} | inf | 102. | 569.255 | {538.132, 602.027} | 458.093 | 0.746277 | newly | 103. | 191.2 | {173., 211.} | 85.6311 | 0.267406 | Reff | 0.913086 | 0.761107 | {0.737846, 0.783779} | 16.6445 | ar | 3516. | 7188.47 | {6948., 7378.} | 104.45 |
| 2 | R0 | {1.32013, 4.45847} | {1.613, 13.6242} | {{1.57463, 12.8503}, {1.66024, 15.1856}} | {22.1849, 205.58} | inf | 44. | 423.337 | {357.457, 521.582} | 862.13 | 0.97609 | newly | 57. | 198.6 | {171., 233.} | 248.421 | 0.538834 | Reff | 0.755774 | 0.729762 | {0.706657, 0.762979} | 4.1513 | ar | 4420. | 7966.18 | {7772., 8151.} | 80.2303 |
| 3 | R0 | {1.31785, 5.36796} | {1.56771, 12.0536} | {{1.51325, 11.0565}, {1.64466, 13.3213}} | {18.9595, 124.547} | inf | 84. | 512.191 | {448.547, 551.256} | 509.751 | 0.778266 | newly | 97. | 273.43 | {245., 301.} | 181.887 | 0.448531 | Reff | 0.69253 | 0.764988 | {0.729869, 0.797803} | 10.4627 | ar | 4942. | 8457.94 | {8064., 8858.} | 71.1441 |
| 4 | R0 | {1.38801, 6.16031} | {1.59715, 15.8165} | {{1.49792, 13.9819}, {1.73255, 20.}} | {15.0677, 156.748} | inf | 76. | 500.581 | {372.103, 713.69} | 558.659 | 0.803286 | newly | 81. | 203.56 | {169., 242.} | 151.309 | 0.394982 | Reff | 0.752718 | 0.716085 | {0.672786, 0.75644} | 5.47687 | ar | 4790. | 7871.37 | {7414., 8446.} | 64.3292 |
| 5 | R0 | {1.27955, 11.57} | {1.51977, 19.532} | {{1.5111, 19.463}, {1.53114, 19.5816}} | {18.7741, 68.816} | inf | 144. | 77.9434 | {75.6834, 79.5624} | 45.8727 | 0.266666 | newly | 92. | 26.13 | {20., 33.} | 71.5978 | 0.554124 | Reff | 0.976553 | 0.612377 | {0.608125, 0.615895} | 37.292 | ar | 3460. | 7145.25 | {7075., 7202.} | 106.51 |
| 6 | R0 | {1.22184, 4.34395} | {1.59152, 16.4553} | {{1.43357, 11.0506}, {1.6615, 18.2958}} | {30.256, 278.81} | inf | 60. | 783.034 | {481.409, 911.456} | 1205.06 | 1.10608 | newly | 80. | 320.46 | {287., 360.} | 300.575 | 0.600774 | Reff | 0.820343 | 0.864722 | {0.817683, 0.925131} | 5.94558 | ar | 3524. | 8304.12 | {7710., 8700.} | 135.645 |
| 7 | R0 | {1.23856, 4.59068} | {1.5174, 15.6368} | {{1.47178, 13.9771}, {1.58607, 20.}} | {22.5131, 240.62} | inf | 59. | 514.532 | {435.971, 653.187} | 772.088 | 0.932295 | newly | 87. | 218.71 | {195., 252.} | 151.391 | 0.39823 | Reff | 0.795403 | 0.797516 | {0.751985, 0.817157} | 2.48359 | ar | 3855. | 7562.11 | {7204., 8114.} | 96.1637 |
| 8 | R0 | {1.34531, 10.6689} | {1.55571, 19.7756} | {{1.51641, 19.3426}, {1.58891, 20.}} | {15.6391, 85.3571} | inf | 248. | 588.993 | {560.039, 619.509} | 137.497 | 0.375339 | newly | 164. | 196.88 | {179., 213.} | 20.1341 | 0.0785598 | Reff | 0.83611 | 0.742118 | {0.713021, 0.767803} | 11.2415 | ar | 4852. | 7445.4 | {7237., 7642.} | 53.4501 |
| 9 | R0 | {1.31775, 5.19226} | {1.60983, 15.7838} | {{1.52954, 13.9295}, {1.70051, 18.2513}} | {22.1651, 203.987} | inf | 73. | 588.106 | {470.608, 726.727} | 705.624 | 0.898135 | newly | 85. | 241.57 | {207., 276.} | 184.2 | 0.450641 | Reff | 0.713693 | 0.734382 | {0.689676, 0.778676} | 4.70593 | ar | 4708. | 8019.33 | {7631., 8524.} | 70.3341 |
| 10 | R0 | {1.25916, 6.89207} | {1.47437, 18.0433} | {{1.41303, 16.5408}, {1.60338, 20.}} | {17.0915, 161.798} | inf | 69. | 508.227 | {400.31, 715.083} | 636.562 | 0.855516 | newly | 65. | 186.48 | {154., 238.} | 186.892 | 0.452011 | Reff | 0.845904 | 0.77694 | {0.767692, 0.792677} | 8.15265 | ar | 3823. | 7366.91 | {6914., 8053.} | 92.6997 |
| 11 | R0 | {1.20377, 5.46566} | {1.53464, 18.5818} | {{1.43453, 14.4101}, {1.62342, 20.}} | {27.4865, 239.974} | inf | 56. | 664.869 | {493.72, 831.808} | 1087.27 | 1.06712 | newly | 56. | 243.11 | {199., 293.} | 334.125 | 0.632968 | Reff | 0.847334 | 0.888054 | {0.819887, 0.94304} | 5.7892 | ar | 3114. | 7770.81 | {7263., 8188.} | 149.544 |
| 12 | R0 | {1.27878, 2.55804} | {1.74336, 13.252} | {{1.70575, 12.7806}, {1.79131, 13.9939}} | {36.3299, 418.052} | inf | 4. | 685.496 | {655.409, 743.443} | 17 037.4 | 2.23319 | newly | 9. | 328.34 | {310., 348.} | 3548.22 | 1.56168 | Reff | 0.778393 | 0.653455 | {0.638491, 0.667499} | 16.0508 | ar | 3930. | 9702.94 | {9536., 9849.} | 146.894 |
| 13 | R0 | {1.41227, 5.45696} | {1.60295, 19.4291} | {{1.50946, 18.6996}, {1.66978, 20.}} | {13.5016, 256.043} | inf | 53. | 582.568 | {531.427, 656.06} | 999.185 | 1.03496 | newly | 51. | 194.83 | {159., 220.} | 282.02 | 0.578891 | Reff | 0.744831 | 0.682231 | {0.638328, 0.724064} | 8.50636 | ar | 4822. | 7751.32 | {7219., 8102.} | 60.7491 |
| 14 | R0 | {1.32917, 7.27593} | {1.57002, 19.3228} | {{1.49772, 18.2125}, {1.63173, 20.}} | {18.1204, 165.571} | inf | 144. | 593.158 | {447.998, 678.684} | 311.915 | 0.610599 | newly | 106. | 202.55 | {167., 228.} | 91.0849 | 0.277705 | Reff | 0.755633 | 0.724954 | {0.686085, 0.760317} | 4.46543 | ar | 4758. | 7461.86 | {7180., 7740.} | 56.8277 |
| 15 | R0 | {1.31629, 9.97481} | {1.55784, 19.6813} | {{1.5089, 19.0356}, {1.58655, 20.}} | {18.3507, 97.3103} | inf | 282. | 728.115 | {690.22, 771.004} | 158.197 | 0.411048 | newly | 192. | 248.19 | {226., 272.} | 29.2656 | 0.110019 | Reff | 0.872437 | 0.82222 | {0.791005, 0.857928} | 5.7559 | ar | 4543. | 7403.51 | {7184., 7638.} | 62.9652 |
| 16 | R0 | {1.36735, 5.74166} | {1.55703, 16.122} | {{1.45688, 13.6346}, {1.67751, 20.}} | {13.8721, 180.79} | inf | 112. | 577.502 | {430.455, 749.059} | 415.626 | 0.701694 | newly | 104. | 233.5 | {203., 261.} | 124.519 | 0.348139 | Reff | 0.782534 | 0.772989 | {0.716311, 0.815156} | 4.24071 | ar | 4516. | 7567.02 | {7033., 8156.} | 67.5602 |
| 17 | R0 | {1.27524, 13.2396} | {1.52933, 19.8686} | {{1.51528, 19.2857}, {1.54768, 20.}} | {19.9251, 50.0695} | inf | 176. | 631.588 | {599.133, 654.143} | 258.857 | 0.554695 | newly | 112. | 215.66 | {197., 235.} | 92.5536 | 0.283548 | Reff | 1.09887 | 0.79948 | {0.782797, 0.810327} | 27.2456 | ar | 3731. | 7191.79 | {7047., 7348.} | 92.7577 |
| 18 | R0 | {1.21884, 7.71545} | {1.54448, 19.6884} | {{1.50938, 19.4389}, {1.57805, 20.}} | {26.717, 155.182} | inf | 93. | 644.517 | {632.97, 675.114} | 593.029 | 0.837799 | newly | 79. | 219.57 | {200., 239.} | 177.937 | 0.442003 | Reff | 0.870374 | 0.788542 | {0.756779, 0.817052} | 9.40188 | ar | 3080. | 7466.4 | {7242., 7673.} | 142.416 |
| 19 | R0 | {1.34879, 6.71464} | {1.628, 16.2198} | {{1.56857, 14.4671}, {1.66599, 18.4705}} | {20.7011, 141.559} | inf | 64. | 489.743 | {394.575, 574.491} | 665.223 | 0.877519 | newly | 49. | 196.99 | {172., 226.} | 302.02 | 0.601532 | Reff | 0.757615 | 0.740284 | {0.71343, 0.783529} | 3.6009 | ar | 4585. | 8326.43 | {8080., 8525.} | 81.6015 |
| 20 | R0 | {1.37284, 5.75036} | {1.55737, 15.1436} | {{1.48477, 12.2707}, {1.62682, 19.3189}} | {13.4413, 163.351} | inf | 131. | 538.053 | {350.771, 753.944} | 310.728 | 0.597503 | newly | 141. | 231.54 | {182., 283.} | 64.2128 | 0.209877 | Reff | 0.7784 | 0.769744 | {0.71974, 0.815121} | 4.37647 | ar | 4750. | 7519.22 | {7095., 8035.} | 58.2994 |
| 21 | R0 | {1.29938, 5.36419} | {1.60222, 14.6486} | {{1.49976, 12.6532}, {1.65675, 16.5775}} | {23.3062, 173.081} | inf | 52. | 487.797 | {362.551, 660.221} | 838.072 | 0.960274 | newly | 62. | 214.28 | {181., 258.} | 245.613 | 0.53476 | Reff | 0.755719 | 0.747397 | {0.708234, 0.808602} | 4.2281 | ar | 4337. | 7926.94 | {7634., 8303.} | 82.7747 |
| 22 | R0 | {1.31882, 7.47626} | {1.52957, 19.6817} | {{1.48797, 19.229}, {1.55957, 20.}} | {15.9805, 163.256} | inf | 105. | 598.338 | {564.032, 639.445} | 469.845 | 0.754898 | newly | 86. | 204.19 | {185., 225.} | 137.43 | 0.374094 | Reff | 0.86897 | 0.768416 | {0.738757, 0.788294} | 11.5717 | ar | 3977. | 7169.91 | {6971., 7396.} | 80.2844 |
| 23 | R0 | {1.26215, 3.46375} | {1.4788, 10.9835} | {{1.46025, 10.4623}, {1.49478, 11.3203}} | {17.1649, 217.099} | inf | 13. | 336.85 | {313.354, 352.782} | 2491.15 | 1.41015 | newly | 14. | 198.57 | {180., 218.} | 1318.36 | 1.15021 | Reff | 0.807776 | 0.774579 | {0.755173, 0.78606} | 4.27209 | ar | 3607. | 7627.13 | {7454., 7771.} | 111.454 |
| 24 | R0 | {1.16712, 4.19464} | {1.52205, 17.0456} | {{1.45298, 13.4878}, {1.67751, 20.}} | {30.4106, 306.366} | inf | 40. | 501.565 | {304.138, 727.572} | 1153.91 | 1.07406 | newly | 56. | 190.48 | {143., 236.} | 240.143 | 0.523768 | Reff | 0.834491 | 0.780519 | {0.750178, 0.82547} | 7.00918 | ar | 3000. | 7697.79 | {7251., 8383.} | 156.593 |
| 25 | R0 | {1.22751, 3.34517} | {1.56508, 15.103} | {{1.47646, 10.7597}, {1.66674, 18.7199}} | {27.5004, 351.488} | inf | 37. | 806.847 | {486.875, 1020.94} | 2080.67 | 1.32174 | newly | 58. | 358.05 | {295., 407.} | 517.328 | 0.787424 | Reff | 0.861344 | 0.906706 | {0.83768, 0.974342} | 6.60888 | ar | 3086. | 8266.87 | {7636., 8717.} | 167.883 |
| 26 | R0 | {1.18747, 5.88083} | {1.57546, 18.3095} | {{1.46452, 14.6062}, {1.71454, 20.}} | {32.674, 211.341} | inf | 33. | 493.67 | {273.907, 673.779} | 1395.97 | 1.15064 | newly | 33. | 174.62 | {119., 228.} | 429.152 | 0.711709 | Reff | 0.772805 | 0.732487 | {0.6912, 0.775854} | 5.45467 | ar | 3614. | 7827.38 | {7356., 8294.} | 116.585 |
| 27 | R0 | {1.29023, 4.76202} | {1.5658, 16.2178} | {{1.49289, 14.1651}, {1.7154, 20.}} | {21.3583, 240.566} | inf | 18. | 490.909 | {352.644, 755.241} | 2627.27 | 1.41978 | newly | 24. | 194.76 | {154., 243.} | 711.5 | 0.903199 | Reff | 0.74614 | 0.727202 | {0.69265, 0.759312} | 3.97091 | ar | 4245. | 7708.86 | {7351., 8372.} | 81.5986 |
| 28 | R0 | {1.2258, 12.9056} | {1.51678, 19.9967} | {{1.50867, 20.}, {1.51838, 20.}} | {23.7377, 54.9458} | inf | 123. | 47.3108 | {45.7565, 47.7708} | 61.5359 | 0.415517 | newly | 72. | 15.13 | {11., 19.} | 78.9861 | 0.687926 | Reff | 0.891402 | 0.598497 | {0.585793, 0.60202} | 32.8589 | ar | 3639. | 7743.9 | {7655., 7820.} | 112.803 |
| 29 | R0 | {1.34702, 6.29049} | {1.5588, 15.7376} | {{1.46191, 13.0883}, {1.70669, 19.6302}} | {15.7222, 150.181} | inf | 82. | 440.95 | {281.905, 614.098} | 437.744 | 0.71158 | newly | 86. | 178.75 | {134., 216.} | 107.849 | 0.310169 | Reff | 0.773998 | 0.725512 | {0.689226, 0.776991} | 6.69891 | ar | 4560. | 7416.57 | {6921., 7991.} | 62.6441 |
| 30 | R0 | {1.37931, 6.78741} | {1.55013, 16.3638} | {{1.47166, 13.7285}, {1.68358, 20.}} | {12.3847, 141.091} | inf | 58. | 455.681 | {319.231, 611.062} | 685.658 | 0.879669 | newly | 51. | 179.15 | {146., 213.} | 251.275 | 0.540692 | Reff | 0.764552 | 0.740394 | {0.687323, 0.782148} | 4.36147 | ar | 4667. | 7466.47 | {6977., 8130.} | 59.9844 |
| 31 | R0 | {1.16062, 4.47529} | {1.61323, 19.9012} | {{1.58952, 20.}, {1.64661, 20.}} | {38.9976, 344.692} | inf | 38. | 883.746 | {842.091, 950.379} | 2225.65 | 1.3661 | newly | 49. | 302.23 | {276., 334.} | 516.796 | 0.78911 | Reff | 0.82288 | 0.896439 | {0.880828, 0.90718} | 8.93926 | ar | 3037. | 8393.87 | {8161., 8670.} | 176.387 |
| 32 | R0 | {1.37647, 18.7122} | {1.52755, 19.9188} | {{1.49593, 19.6805}, {1.56253, 20.}} | {10.976, 6.44815} | inf | 359. | 69.1761 | {62.0546, 76.9281} | 80.7309 | 0.71669 | newly | 137. | 22.3 | {17., 27.} | 83.7226 | 0.796203 | Reff | 0.886034 | 0.589239 | {0.583142, 0.594885} | 33.497 | ar | 4824. | 7548.66 | {7397., 7682.} | 56.4813 |
| 33 | R0 | {1.35084, 6.08841} | {1.63058, 15.7936} | {{1.52145, 14.2303}, {1.73278, 20.}} | {20.7086, 159.404} | inf | 48. | 510.041 | {409.019, 679.344} | 962.585 | 1.01656 | newly | 49. | 207.46 | {169., 251.} | 323.388 | 0.622249 | Reff | 0.722834 | 0.699344 | {0.649358, 0.753642} | 5.46076 | ar | 4759. | 8068.76 | {7555., 8512.} | 69.5474 |
| 34 | R0 | {1.36912, 6.316} | {1.567, 19.2346} | {{1.45725, 15.1602}, {1.64301, 20.}} | {14.453, 204.538} | inf | 145. | 668.588 | {486.414, 761.35} | 361.095 | 0.657797 | newly | 140. | 229.22 | {190., 261.} | 63.7286 | 0.210964 | Reff | 0.820103 | 0.787662 | {0.738079, 0.840105} | 5.01128 | ar | 4537. | 7412.1 | {6958., 7772.} | 63.3701 |
| 35 | R0 | {1.35831, 7.09038} | {1.63457, 15.6353} | {{1.56473, 14.1032}, {1.79883, 20.}} | {20.3385, 120.515} | inf | 73. | 460.761 | {353.266, 651.987} | 531.179 | 0.786235 | newly | 59. | 189.07 | {152., 232.} | 220.458 | 0.499825 | Reff | 0.752504 | 0.712203 | {0.681219, 0.746334} | 5.74599 | ar | 4667. | 8128.35 | {7826., 8897.} | 74.1665 |
| 36 | R0 | {1.28315, 5.89938} | {1.63642, 15.5724} | {{1.56453, 13.4189}, {1.70645, 19.5171}} | {27.5315, 163.967} | inf | 40. | 555.407 | {423.575, 793.549} | 1288.52 | 1.13156 | newly | 43. | 231.22 | {199., 265.} | 437.721 | 0.72779 | Reff | 0.715356 | 0.753281 | {0.721763, 0.791714} | 5.72258 | ar | 4460. | 8392.8 | {8063., 8834.} | 88.1794 |
| 37 | R0 | {1.19303, 2.75113} | {1.46303, 14.075} | {{1.37183, 10.6281}, {1.58961, 19.1389}} | {22.6315, 411.607} | inf | 5. | 507.257 | {359.403, 714.923} | 10 045.1 | 1.98507 | newly | 9. | 238.88 | {210., 269.} | 2554.22 | 1.42205 | Reff | 0.839297 | 0.87857 | {0.848758, 0.931502} | 4.797 | ar | 3142. | 7267.87 | {6678., 8070.} | 131.313 |
| 38 | R0 | {1.36033, 7.8088} | {1.58565, 16.8761} | {{1.47486, 13.9864}, {1.74122, 20.}} | {16.5639, 116.116} | inf | 165. | 588.062 | {400.425, 768.718} | 256.401 | 0.536882 | newly | 127. | 227.96 | {184., 272.} | 79.4961 | 0.249123 | Reff | 0.778789 | 0.778033 | {0.71748, 0.817654} | 3.91819 | ar | 4941. | 7883.41 | {7255., 8592.} | 59.5509 |
| 39 | R0 | {1.27353, 3.70845} | {1.6769, 17.8823} | {{1.63479, 13.8698}, {1.6999, 19.2372}} | {31.6735, 382.205} | inf | 7. | 637.033 | {475.248, 696.883} | 9000.46 | 1.95485 | newly | 14. | 236.2 | {216., 255.} | 1587.14 | 1.22623 | Reff | 0.800796 | 0.782407 | {0.75399, 0.800058} | 2.4184 | ar | 3725. | 8719.72 | {8481., 8909.} | 134.086 |
| 40 | R0 | {1.19983, 12.1486} | {1.48711, 19.609} | {{1.47093, 19.5113}, {1.49982, 19.6609}} | {23.9432, 61.4094} | inf | 140. | 38.0131 | {36.7519, 38.8793} | 72.8478 | 0.566345 | newly | 71. | 12.43 | {9., 16.} | 82.493 | 0.769765 | Reff | 0.902632 | 0.600141 | {0.589098, 0.604565} | 33.5121 | ar | 3619. | 7293.2 | {7187., 7366.} | 101.525 |
| 41 | R0 | {1.20935, 2.67169} | {1.66822, 13.4452} | {{1.6585, 13.179}, {1.67865, 13.6958}} | {37.9435, 403.245} | inf | 0.1 | 717.826 | {698.524, 744.546} | 717 726. | 3.8559 | newly | 0.1 | 348.84 | {328., 370.} | 348 740. | 3.54217 | Reff | 0.794664 | 0.74046 | {0.733824, 0.746361} | 6.82102 | ar | 3429. | 9258.68 | {9080., 9383.} | 170.011 |
| 42 | R0 | {1.23313, 4.74781} | {1.54484, 19.0216} | {{1.47445, 18.0165}, {1.61511, 20.}} | {25.2776, 300.639} | inf | 65. | 549.172 | {458.17, 665.195} | 744.879 | 0.918414 | newly | 90. | 190.79 | {158., 227.} | 111.989 | 0.32133 | Reff | 0.791546 | 0.755065 | {0.714361, 0.793314} | 5.23504 | ar | 3835. | 7342.92 | {7000., 7651.} | 91.4712 |
| 43 | R0 | {1.26105, 2.39713} | {1.73701, 17.676} | {{1.65776, 16.096}, {1.80496, 19.1847}} | {37.7428, 637.382} | inf | 1. | 910.332 | {799.84, 1006.98} | 90 933.2 | 2.95699 | newly | 1. | 341.55 | {314., 367.} | 34 055. | 2.53255 | Reff | 0.791561 | 0.752631 | {0.727861, 0.77425} | 5.2893 | ar | 3723. | 9890.3 | {9514., 10 198.} | 165.654 |
| 44 | R0 | {1.36948, 8.88006} | {1.56706, 19.5081} | {{1.51799, 18.8521}, {1.61796, 20.}} | {14.4277, 119.685} | inf | 166. | 691.849 | {573.894, 759.736} | 316.776 | 0.616618 | newly | 127. | 237.18 | {204., 264.} | 86.7559 | 0.268551 | Reff | 0.844011 | 0.790324 | {0.723122, 0.83439} | 6.50524 | ar | 4437. | 7435.38 | {7100., 7799.} | 67.5767 |
| 45 | R0 | {1.34882, 3.3791} | {2.02047, 15.1048} | {{1.98977, 14.2749}, {2.04865, 17.8452}} | {49.7954, 347.007} | inf | 6. | 893.476 | {822.205, 1121.07} | 14 791.3 | 2.17057 | newly | 9. | 376.74 | {355., 405.} | 4086. | 1.62114 | Reff | 0.755069 | 0.638797 | {0.616128, 0.653663} | 15.399 | ar | 4422. | 10 191. | {10 052., 10 360.} | 130.46 |
| 46 | R0 | {1.23774, 4.34917} | {1.58784, 15.3924} | {{1.51313, 13.0771}, {1.6933, 18.3859}} | {28.2855, 253.916} | inf | 30. | 640.71 | {502.124, 840.886} | 2035.7 | 1.32142 | newly | 38. | 274.02 | {238., 320.} | 621.105 | 0.855061 | Reff | 0.787698 | 0.819898 | {0.76497, 0.88082} | 5.69749 | ar | 3685. | 8042.18 | {7631., 8498.} | 118.241 |
| 47 | R0 | {1.34327, 4.78811} | {1.69585, 14.9863} | {{1.62351, 12.8542}, {1.75441, 18.6326}} | {26.2481, 212.99} | inf | 76. | 665.312 | {489.23, 973.583} | 775.41 | 0.927624 | newly | 94. | 288.91 | {239., 349.} | 207.351 | 0.482933 | Reff | 0.773992 | 0.762743 | {0.715162, 0.806647} | 3.94626 | ar | 4517. | 8385.24 | {8046., 8805.} | 85.6374 |
| 48 | R0 | {1.24366, 4.25814} | {1.54836, 16.4128} | {{1.46291, 13.9007}, {1.66362, 20.}} | {24.5001, 285.445} | inf | 38. | 538.438 | {382.06, 814.94} | 1316.94 | 1.12963 | newly | 50. | 213.61 | {179., 276.} | 327.22 | 0.623813 | Reff | 0.803653 | 0.795829 | {0.73786, 0.849193} | 3.89793 | ar | 3678. | 7584.97 | {7055., 8288.} | 106.225 |
| 49 | R0 | {1.36017, 7.5415} | {1.53643, 19.4083} | {{1.48033, 18.6535}, {1.63357, 20.}} | {12.9588, 157.353} | inf | 152. | 572.551 | {457.014, 622.137} | 276.678 | 0.573713 | newly | 140. | 195.87 | {166., 219.} | 39.9357 | 0.14364 | Reff | 0.945454 | 0.737178 | {0.698886, 0.758168} | 22.0292 | ar | 4178. | 7488.6 | {7239., 7735.} | 79.2389 |
| 50 | R0 | {1.25333, 6.63578} | {1.68331, 19.1766} | {{1.58056, 18.6466}, {1.73217, 19.9219}} | {34.3072, 188.988} | inf | 49. | 541.589 | {474.522, 659.802} | 1005.28 | 1.04003 | newly | 43. | 187.87 | {165., 226.} | 336.907 | 0.637051 | Reff | 0.846499 | 0.739331 | {0.694843, 0.786474} | 12.6601 | ar | 3401. | 8566.03 | {8324., 8773.} | 151.868 |
